# An Algorithmic Approach to Compute the Effect of Non-Radiative Relaxation Processes in Photoacoustic Spectroscopy

**DOI:** 10.1016/j.pacs.2022.100371

**Published:** 2022-05-13

**Authors:** Max Müller, Thomas Rück, Simon Jobst, Jonas Pangerl, Stefan Weigl, Rudolf Bierl, Frank-Michael Matysik

**Affiliations:** a Sensorik-ApplikationsZentrum (SappZ), Regensburg University of Applied Sciences, 93053 Regensburg, Germany; b Institute of Analytical Chemistry, Chemo, and Biosensors, University of Regensburg, 93053 Regensburg, Germany

**Keywords:** Photoacoustic spectroscopy, Relaxation effects, Molecular energy transitions, Algorithm simulation

## Abstract

Successful transfer of photoacoustic gas sensors from laboratory to real-life applications requires knowledge about potential cross-sensitivities towards environmental and gas matrix changes. Multi-dimensional calibration in case of cross-sensitivities can become very complex or even unfeasible. To address this challenge, we present a novel algorithm to compute the collision based non-radiative efficiency and phase lag of energy relaxation on a molecular level (CoNRad) for photoacoustic signal calculation. This algorithmic approach allows to calculate the entire relaxation cascade of arbitrarily complex systems, yielding a theoretical photoacoustic signal. In this work the influence of varying bulk compositions, i.e. nitrogen (N_2_), oxygen (O_2_) and water (H_2_O) on the photoacoustic signal during methane (CH_4_) detection is demonstrated. The applicability of the algorithm to other photoacoustic setups is shown exemplary by applying it to the relaxational system investigated in [1]. Hayden et al. examined the effect of water on photoacoustic carbon monoxide (CO) detection.

## 1 Introduction

In the last 20 years, scientific and commercial interest in photoacoustic (PA) gas sensors has increased immensely. This burgeoning attention becomes apparent by the number of publications related to the subject over the years. While in 2000 the number of annual publications regarding “photoacoustic sensors” was fairly modest with 140 according to the literature database *Dimensions.ai*, it grew sharply to 799 in 2020 [2]. In previous works, the focus was often solely on the advantages of photoacoustic gas sensors, e.g. high spectral selectivity, high sensitivity and the great potential for miniaturization, in order to demonstrate the capability for mobile trace gas analysis. As a result, miniaturized and low-cost PA sensor systems with remarkable limits of detection (LoD) in the parts per billion (ppbV) or even parts per trillion (pptV) range have been published [3], [4], [5], [6], [7], [8], [9], [10], [11], [12], [13], [14], [15], [16], [17], [18], [19], [20]. However, in recent years the major disadvantage of photoacoustics, namely the PA-signal dependency on a changing bulk composition due to relaxation-based energy dissipation has been increasingly addressed and analyzed by the scientific community [1], [4], [9], [13], [14], [21], [22], [23], [24], [25]. The photoacoustic signal results from molecular collisions converting internal energy states into kinetic energy of translation. By studying recent literature dealing with the importance of molecular relaxation in photoacoustics, it becomes noticeable that a wealth of the published work is prone to inadequacies in the assumptions made. The cascade of relaxation processes is often oversimplified, e.g. by hypothesizing two-level systems, which can only be applied if the initially excited state is the lowest one, i.e. no species of the composition exhibits an intermediate state between the excited one and ground state [26], and by disregarding vibrational-vibrational (VV) energy transfer processes. However, to be able to develop photoacoustic sensors that provide reliable analyte readings even in complex, frequently altering bulk mixtures, a comprehensive understanding of the phenomenon of molecular collisional relaxation is inevitable. Hunter et al. [26] already presented a universal relaxation model based on reaction kinetics in 1974 to calculate the population densities of all energy states of every molecule involved in PA-signal generation as well as the complex heat production rates of the separate relaxation paths finally yielding the overall heat production and photoacoustic phase lag. In a previous work we implemented the underlying mathematical correlations of Hunter et al. into a MATLAB script by predefining the route of relaxation in terms of mid-infrared methane excitation in a gas matrix containing nitrogen, oxygen and water and compared the simulations with our measurement results [9]. However, due to restrictions in the measurement setup in [9], we were incapable of measuring the water-induced signal increase in detail, since the smallest adjustable water concentration already resulted in 100% accelerated relaxation. For this reason, we modified our gas mixing system by integrating a simple self-designed humidity generator (see chapter 2.5., Fig. 3). This setup enables humidification of the sample gas in much smaller increments, which allows for a more detailed investigation of amplitude and phase characteristics.

The development of CoNRad was based on the fundamental physical understanding of relaxation processes. CoNRad autonomously identifies the entire relaxation cascade of a given system. Based on this, the overall relaxation efficiency considering mutually competing energy transitions is calculated, yielding a completely theoretically derived photoacoustic signal. Thus, potential influences on the PA signal resulting from relaxation phenomena can be determined not only qualitatively but also quantitatively. As a result, our approach prevents erroneous conclusions that might otherwise be drawn due to a lack of distinction between various physical effects.

## 2 Methods

### 2.1 Photoacoustic signal generation

The first step in order to describe the photoacoustic signal mathematically is to establish a formula for the time dependent heat production rate per volume $\dot{H}\left(t\right)$ with unit Js^−1^m^−3^.

(1) $$\dot{H}(t)=\left[\nu_{A}\right]\left(t\right)\frac{\left({\text{hc}}_{0}{\bar{\nu }}_{\text{Ph}}\right)}{\tau_{\text{A}}}={\dot{H}}_{0}e^{i(\omega t-\Phi_{\text{A}})}$$

(2) $${\dot{H}}_{0}=\frac{\rho_{\text{A}}\sigma_{\text{A}}\left({\bar{\nu }}_{\text{Ph}}\right)}{\pi r_{\text{b}}^{2}}\cdot \frac{1}{\sqrt{1+{\left(\omega \tau_{\text{A}}\right)}^{2}}}$$

Therein $\rho_{A}$ is the volume number density of the analyte A, $\left[\nu_{A}\right](t)$ is the time dependent population density of the excited analyte state,^2^ h is the Planck constant, ${\text{c}}_{0}$ the speed of light in vacuum, $\sigma_{A}\left({\bar{\nu }}_{\mathrm{Ph}}\right)$ the absorption cross section in m² at the emitted wavenumber ${\overset{®}{\nu }}_{\mathrm{Ph}}$ and $\tau_{A}$ is the non-radiative relaxation lifetime of the excited state of the analyte. The phase lag $\phi_{A}=\arctan\left(\omega \tau_{A}\right)$ represents the time that is needed for PA signal generation, i.e. the duration from photon absorption to local heat input. The laser beam radius is designated to $r_{b}$ and its optical power $P_{0}$ is modulated with an angular frequency ω=2πf.

In terms of photoacoustic spectroscopy (PAS), the combined solution of the Navier-Stokes equation, the thermal diffusion equation and the mass-density continuity equation yields a dampened wave equation for the photoacoustic sound pressure $p_{a}$ [5], [9], [27]. This wave equation provides the correlation between heat input and acoustic pressure. Regardless of the photoacoustic measurement setup, the photoacoustic pressure $p_{a}$ can generally be defined as a function of the relaxation induced heat production rate $\;\dot{H}(t)$ (refer to equations (1) and (2))

(3) $$p_{a}\propto \frac{\rho_{A}\sigma_{A}\left({\bar{\nu }}_{\mathrm{ph}}\right)P_{0}}{\pi {r_{b}}^{2}}.\frac{1}{\underset{\left|{\vec{\varepsilon }}_{\mathrm{relax}}\right|}{\underbrace{\sqrt{1+{\left(\omega \tau_{A}\right)}^{2}}}}}$$

with $\left|{\overset{⃑}{\epsilon }}_{\mathrm{relax}}\right|$ representing the efficiency of total non-radiative relaxation.

If, for instance, the lowest energy state $\nu_{1,A}$ of a molecule A, with its time dependent population density written as $\left[\nu_{1,A}\right]\left(t\right)$, is excited by laser radiation that may exclusively relax to the vibronic ground state, equation can be written as,

(4) $${\dot{H}}_{\nu_{1,\text{A}}}\left(t\right)=\left[\nu_{1,\text{A}}\right]\left(t\right)\cdot \frac{\left({\text{hc}}_{0}{\bar{\nu }}_{1,\text{A}}\right)}{\tau_{\nu_{1,\text{A}}}}=\left[\nu_{1,\text{A}}\right]\left(t\right)\cdot \left(k_{\nu_{1,\text{A}}}\cdot {\mathrm{hc}}_{0}{\bar{\nu }}_{1,A}\right)$$

where the relaxation time is substituted with its inverse relaxation rate $k_{\nu_{1,A}}=1/\tau_{\nu_{1,A}}$. However, equation only accounts for the simplest scenario of exclusive vibrational-translational (VT) relaxation of the initially excited energy level to the ground state $\nu_{0}$. Inter – or intramolecular energy transitions (VV relaxation), in which some of the vibronic energy is not released as translational energy are neglected. Barreiro et al. as well as our group demonstrated in previous works, that these simplifications cannot be applied to many real applications [9], [24], [25]. Hunter et al. modified equation (4) for arbitrarily complex relaxation systems [26]. A brief derivation of the formula established by Hunter et al. is provided in the appendix A. For a more in-depth explanation refer to [26]. Hence, the population densities of the individual energy states not only depend on the quantity of initially excited analyte molecules, but also on the entire relaxation process [26]. Each energy transition contributes an individual phase and amplitude to the overall photoacoustic signal vector.

Real-life applications of photoacoustic sensors are often subject to complex and varying gas compositions (e.g. relative humidity in ambient air or carbon dioxide (CO_2_) in exhaled breath), which significantly increases the number of collision reactions to be considered compared to simple gas matrices containing only the analyte diluted in nitrogen. However, it is impractical to manually compute the entire relaxation cascade, considering a multitude of mutually influencing energy transitions. Therefore, we developed an algorithm (CoNRad) that provides an elegant solution to this problem.

### 2.2 The algorithm (CoNRad)

CoNRad, an algorithm to compute the collision based non-radiative efficiency and phase lag of energy relaxation on a molecular level, implemented in Python programming language, requires all energy states and all possible vibronic energy transfer reactions of the system for cascade computation, which often demands elaborate literature research. Those reactions are tabulated and linked to a relaxation rate, which must also be searched for in literature. The main difference between our approach and Hunter’s et al. is that we take into account the heat released for each single reaction, while Hunter et al. consider the cumulative heat production of all reactions that contribute to one specific change of states [26]. Therefore, CoNRad can be described as a reaction-based approach, whereas Hunter’s et al. calculations are rather based on changes in density of states.

**Fig. 1:**
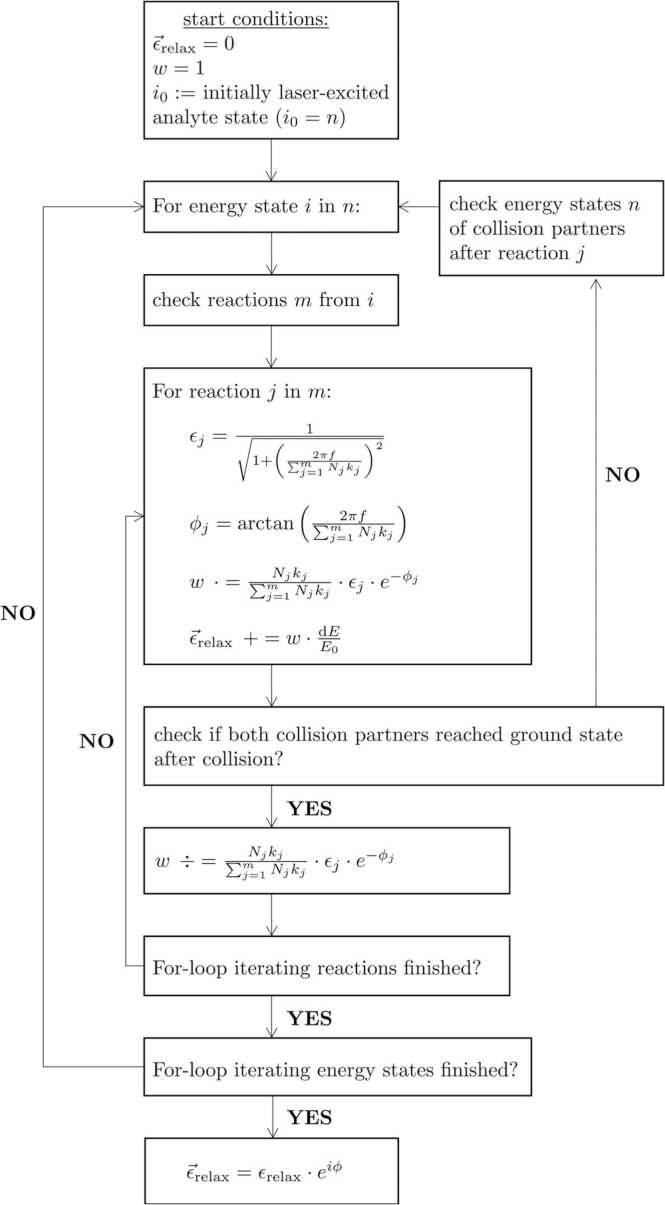
Programming flowchart of CoNRad for computing relaxation dependent PA signals.

Fig. 1 illustrates a simplified programming flowchart of CoNRad. As a prerequisite for cascade calculation, the initially laser-excited analyte state $i_{0}$ must be predefined. Further start conditions are ${\overset{⃑}{\epsilon }}_{\mathrm{relax}}=0$ and weighting w=1 specifying the complex efficiency of overall relaxation and a weighting factor, respectively. After computation, the absolute value $\left|{\overset{⃑}{\epsilon }}_{\text{relax}}\right|$ of this complex efficiency is the percentage of energy that is introduced by the laser and converted into kinetic energy of translation, thus contributing to the PA signal. The phase angle of ${\overset{⃑}{\epsilon }}_{\mathrm{relax}}$ quantifies the overall phase lag of signal generation caused by relaxational delay. The weighting factor w, in turn, represents the memory of the recursive function, which performs the calculations (see Fig. 1).

By triggering the algorithm, the table of reactions is scanned for those reactions m, that emanate from the initial state $i_{0}$. These reactions are iteratively (index j) executed until all of them have been processed. The mathematical representations in Fig. 1 specify the contribution of individual reactions to the efficiency of relaxation $\epsilon_{j}$ as well as to the phase lag due to relaxational delay $\phi_{j}.$ Both terms involve the product of relaxation rate $k_{j}$ and the volume ratio of the collision partner $N_{j}$ summarized over all reactions m, which reveals their competitive nature. To quantify heat production, the term $dE/E_{0}$ considers the energy of product states minus reactant states dE as well as the energy of the initially laser-excited state $E_{0}$. Whilst iterative execution, every reaction j is checked to see whether both collision products have reached the ground state already. If not, the excited product states are used as input value to recursively trigger the algorithm from the start, i.e. looking up all reactions m that emanate from state i. If, on the other hand, both collisional products have reached the ground state and both *For*-loops iterating energy states and reactions have run through, the calculation is completed.

The resulting relaxation efficiency ${\overset{⃑}{\epsilon }}_{\mathrm{relax}}$ is multiplied with the cell constant $C_{\mathrm{cell}}$, containing the quality factor Q, the resonance frequency $\omega_{\mathrm{res}}$, the ratio of acoustical resonator length and volume $L_{r}/V_{r}$ and the decremented adiabatic exponent of the measurement gas $\left(\gamma -1\right).$ Further, the optical power $P_{0}$, the microphone sensitivity in µV mbar^−1^ and a refinement factor $C_{\mathrm{corr}}$ are also considered. This factor was found to be 0.865 and kept constant for every data point and every measurement series. The purpose of $C_{\mathrm{corr}}$ is to transfer the simulated data from CoNRad to the measured photoacoustic voltage, by accounting for a multitude of potential non-ideal conditions in the measurement setup, e.g. non-ideal (< 100%) light to sound coupling.

**Fig. 2:**
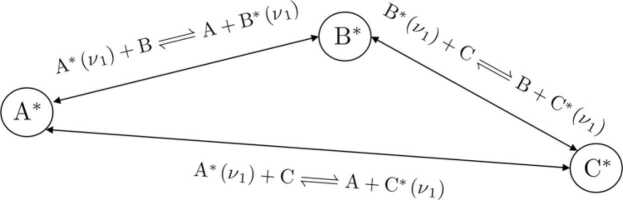
Example of three excited states $A^{*}\left(\nu_{1}\right)$, $B^{*}\left(\nu_{1}\right)$ and $C^{*}\left(\nu_{1}\right)$ forming a circular reference.

It should be noted that only the general function principle of the algorithm is discussed within this context. More sophisticated routines had to be developed to account for reactions which depend on each other or even form circular references (refer to Fig. 2). Particularly, methane, oxygen and water form such circular transitions (see Figure 7, k_6–11_), which are discussed in detail in chapter 3.4.

### 2.3 Experimental setup

**Fig. 3:**
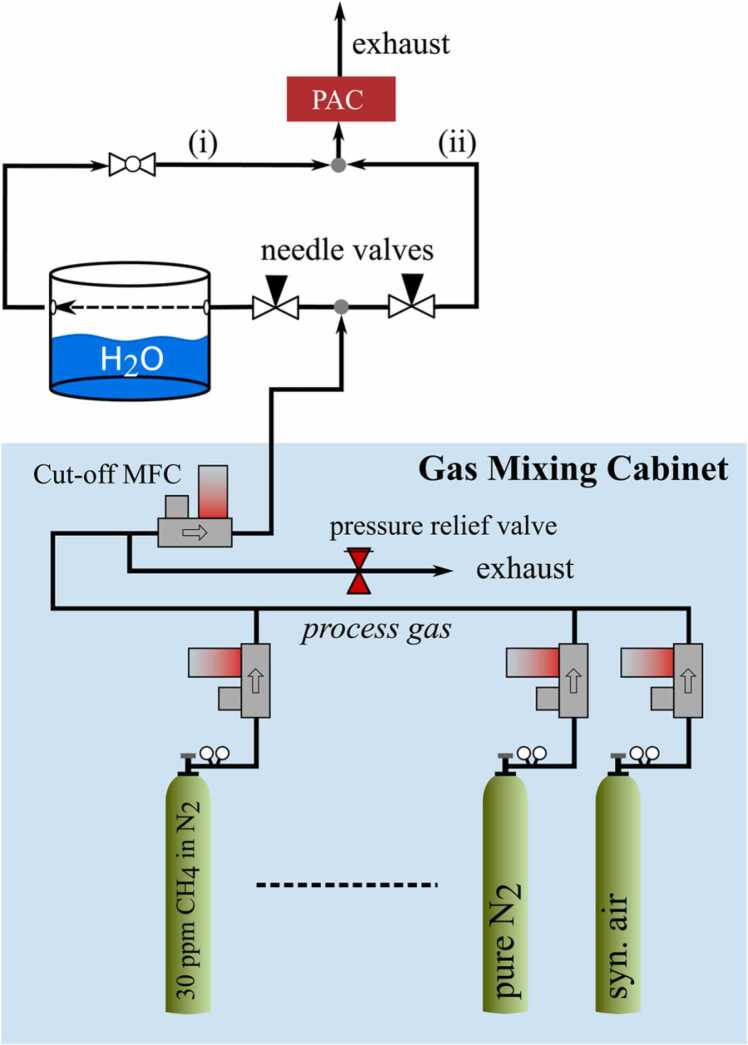
Simplified block diagram of the gas mixing unit of the laboratory test bench. Gas stream (i) indicates the humidified gas, (ii) represents the dry gas flow.

The experimental setup used for this work is almost identical to [9]. Only the gas mixing system was modified, see Fig. 3. The commercially available humidity generator used in our previous work [9] was substituted by a simple self-designed humidity generator, which consists of a temperature-controlled aluminum tank filled with water. The measurement gas can be directed into the photoacoustic measurement cell (PAC) via a tee fitting connected to the humidified gas path (Fig. 3, (i)) and the dry path (Fig. 3, (ii)). It should be mentioned that the process gas is humidified only by the gas phase inside the aluminum tank, as it does not pass through the liquid water. The fraction of the process gas that is humidified can be adjusted manually, using two needle valves. The final humidity content present in the PAC cell is measured by a BME280 (Bosch, Germany). When bypassing gas path (i) the dry gas mixture enters the PAC directly.

Analogous to [9], we use the same interband cascade laser (ICL) diode mounted into a TO66 package, emitting at 3368.8 nm (2968.4 cm^−1^) for methane ($\sigma_{{\mathrm{CH}}_{4}}=4.9\cdot 10^{-19}{\mathrm{cm}}^{2}{\mathrm{mol}}^{-1}$ at ambient pressure and 40 °C) detection.

## 3 Results and discussion

To emphasize the relevance of VV transitions when interpreting photoacoustic measurements, in chapter 3.1. the simplest possible theoretical Jablonsky diagram of a ternary system is discussed. In this theoretical relaxation scenario the analyte molecule A can only relax via VT transitions. Molecule C is successively added to the measurement matrix by increments of 0.1%V and up to a maximum volume ratio of *N*_C_ = 4.9%V. The analyte volume ratio remains constant with *N*_A_ = 15 ppmV for each gas composition. Hence, the volume ratio of molecule B is *N*_B_ = 1 – *N*_A_ – *N*_C_.

The following chapters deal with the different effects of oxygen (chapter 3.2.), water (chapter 3.3.) and the combined effect of oxygen and water (chapter 3.4.) on the mid-IR (2968 cm^−1^) photoacoustic detection of methane, to verify CoNRad for real-life scenarios. Chapter 3.5. compares the data provided by Hayden et al. [1] with those obtained from CoNRad for the mid-IR (2180 cm^−1^) detection of carbon monoxide, with regards to water induced relaxational phenomena.

### 3.1 Vibrational-translational relaxation

**Fig. 4:**
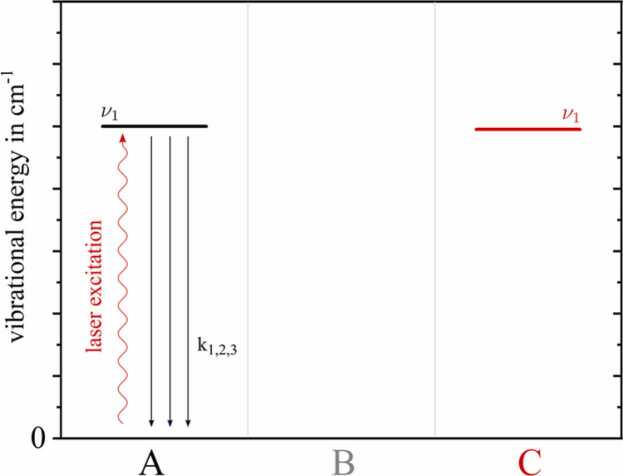
Theoretical Jablonski diagram for non-radiative relaxation, only considering VT transitions.

The theoretical Jablonski diagram shown in Fig. 4 describes the simplest case of non-radiative relaxation of a ternary system. The y-axis represents the energy of the respective vibrational states in cm^−1^, the x-axis subdivides the participating molecules A, B and C. Although C shows an energetically comparable vibrational state ($\nu_{1,C}$) to the initially excited state $\nu_{1,A}$, in this example $\nu_{1,A}$ can only relax directly to the ground state via classical VT relaxation. Molecule B shows no relevant vibrational state in the displayed region.

Table 1 summarizes the respective energy transition reactions together with the reaction rates $k_{1,2,3}$ that are shown in Fig. 4.

**Table 1:** Individual reactions and their reaction ratios $k_{1,2,3}$ for the discussed Jablonski diagram (Fig. 4).

Reaction		k in s^−1^ atm^−1^
(R_1_)	$A^{*}\left(\nu_{1}\right)+A\overset{k_{1}}{\to }A+\mathrm{A;}\;\mathrm{dE}=-E_{\nu_{1,A}}$	$1\cdot 10^{6}$
(R_2_)	$A^{*}\left(\nu_{1}\right)+B\overset{k_{2}}{\to }A+B;\;dE=-E_{\nu_{1,A}}$	$5\cdot 10^{3}$
(R_3_)	$A^{*}\left(\nu_{1}\right)+C\overset{k_{1}}{\to }A+C;\;dE=-E_{\nu_{1,A}}$	$1\cdot 10^{2}\;1\cdot 10^{3}\;1\cdot 10^{4}\;1\cdot 10^{5}\;1\cdot 10^{6}$

**Fig. 5:**
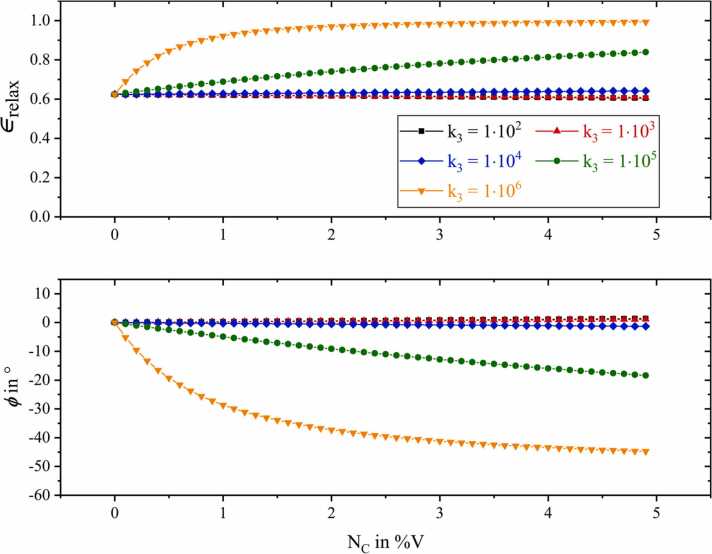
Simulated relative photoacoustic signal amplitudes (upper graphs) and corresponding phase lags (lower graphs) for the theoretical relaxation cascade shown in Fig. 4. The reaction rate of VT relaxation of A colliding with C (Table 1, R_3_) is varied from $1\cdot 10^{2}$ to $1\cdot 10^{6}$ in logarithmic steps.

The rates of (R_1_) and (R_2_) were kept constant in this example, since the influence of molecule C on the VT relaxation is of particular interest. For this purpose, five scenarios (Fig. 5) were simulated starting with a comparatively slow VT energy transition $k_{3}=1\cdot 10^{2}$ of A with C, logarithmically increasing up to $k_{3}=1\cdot 10^{6}$.

As this scenario is purely hypothetical, for the purpose of simplicity the resonance frequency was kept constant at $\omega_{\mathrm{res}}=2\pi \cdot 1000\mathrm{Hz}$. In real applications, gas composition induced resonance frequency variations must be accounted for, too. However, the influence of frequency shifts usually affects the photoacoustic signal only in a minor way compared to the influence of relaxation rates. Furthermore, in this subchapter the additional calculations mentioned in chapter 2.2., i.e. considering $C_{\mathrm{cell}}$, $S_{\mathrm{mic}}$ and $C_{\mathrm{corr}}$ are omitted.

In Fig. 5 the upper graph displays the overall efficiency of relaxation $\epsilon_{\mathrm{relax}}$, being equivalent to the relaxation dependent photoacoustic magnitude. In addition, the graph below represents the corresponding relative phase lag ϕ with reference to the photoacoustic phase of the first simulation point. A relative photoacoustic signal amplitude of $\epsilon_{\mathrm{relax}}=1$ would be equivalent to 100% relaxation of all states actively involved in the cascade, yielding no relaxation losses. In this particular two-level scenario (see Fig. 4) $\epsilon_{\mathrm{relax}}=1$ would correspond to 100% relaxation of $\nu_{1,A}$.

Referring to Fig. 5, the first amplitude value is $\epsilon_{\mathrm{relax}}=0.62$. This implies that $N_{A}=15\mathrm{ppmV}$ being only diluted with molecules of type B, i.e. no C is present, and with the relaxation rates given in Table 1, 38% of the initially excited analyte molecules A are not able to relax in time, thus not releasing any extra kinetic energy into the system. Regarding photoacoustic signal generation, this percentage is lost.

By successively adding C to the composition, five different amplitude and phase characteristics are obtained for the respective relaxation rate $k_{3}$. The most distinct effect, both in amplitude and phase, can be observed for very rapid VT relaxation of A colliding with C $\left(k_{3}=1\cdot 10^{6}\right)$, represented by the orange downward pointing triangles in Fig. 5. At the maximum simulated volume ratio of $N_{C}=4.9\%V$%V, the relative photoacoustic amplitude corresponds to $\epsilon_{\mathrm{relax}}=0.99$. Conversely, the relaxation-related signal loss is only around 1%. The exponential signal increase for $k_{3}=1\cdot 10^{6}$ is accompanied by a significant phase shift $\Delta \phi \left(N_{C}=4.9\%V\right)=-44.7{}^{\circ}$. However, once the VT relaxation of A with C is only decreased by a factor of 10 $\left(k_{3}=1\cdot 10^{5}\right)$, this exponential effect in amplitude and phase becomes an almost linear characteristic (green circle data points in Fig. 5). Even slower relaxation rates hardly show any significant influence on the photoacoustic signal anymore.

**Fig. 6:**
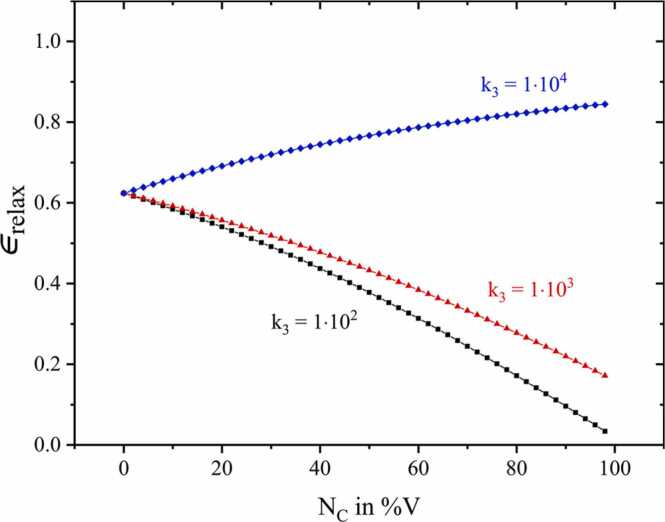
Relative photoacoustic signal amplitude for higher C concentrations $\left(N_{\text{C, max}}=99\%\text{V}\right)$ simulated for $k_{3}=1\cdot 10^{4}$ (blue rhombus), $k_{3}=1\cdot 10^{3}$ (red triangles) and $k_{3}=1\cdot 10^{2}$ (black squares).

By simulating higher concentrations of C up to almost 100%V, it becomes clear that moderately fast VT transitions of A with C $\left(k_{3}=1\cdot 10^{4}\right)$ still result in an increasing amplitude, albeit with a modest slope (see Fig. 6). On the contrary, the decelerating effect of the improbable VT relaxation $\left(k_{3}\leq 1\cdot 10^{3}\right)$ of A with C dominates for higher C concentrations, even yielding a further amplitude decrease due to relaxation losses.

Fig. 5 and Fig. 6 demonstrate that assuming exclusively VT relaxation of the analyte, decelerating relaxation processes fail to explain exponential amplitude losses. Those amplitude characteristics are, however, published in literature [9], [28] and addressed in detail in chapter 3.2.

### 3.2 The effect of oxygen on the photoacoustic detection of methane

**Fig. 7:**
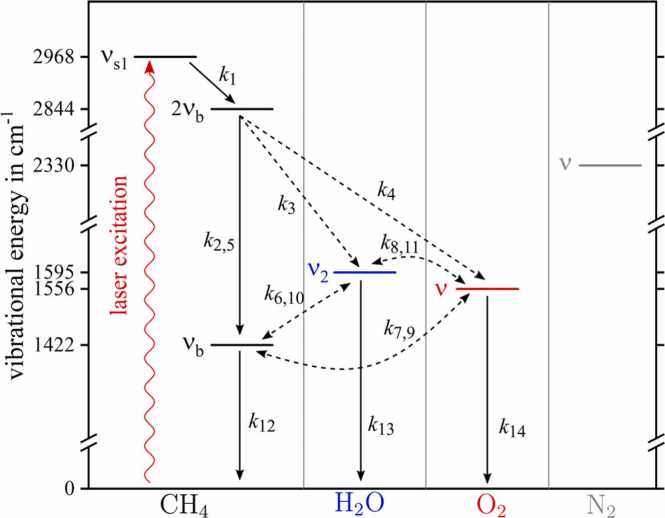
Complete Jablonsky diagram of mid-IR laser excitation of methane, followed by collision based non-radiative relaxation processes with methane, water, oxygen and nitrogen. Intramolecular energy transitions or VT-transitions are indicated as solid arrows. Dashed arrows represent intermolecular transitions.

This chapter addresses the interpretations of empirically data obtained by photoacoustic detection of traces of methane in mixtures of nitrogen and oxygen. Fig. 7 provides the complete Jablonsky diagram of the laser excitation of methane and subsequent collisional relaxation processes that can occur in such mixtures, further including water, which influence is addresses in chapter 3.3. and 3.4. Analogous to [9] some vibrational modes of methane are summarized for simplicity. Mode $\nu_{b}=1422\;{\mathrm{cm}}^{-1}$ (dyad) includes the $\nu_{2}$ and $\nu_{4}$ bending modes, the harmonics of the bending modes ${2\nu }_{2}$, ${2\nu }_{4}$ and $\nu_{4}+\nu_{2}$ are combined into $2\nu_{b}=2844\;{\mathrm{cm}}^{-1}$ and $\nu_{{\text{s}}_{1}}=2968\;{\mathrm{cm}}^{-1}$ represents the two stretching modes $\nu_{1}$ and $\nu_{3}$. Fig. 7 illustrates the purely passive role of nitrogen in the complete relaxation process of methane, i.e. no involvement in any VV transitions.

**Fig. 8:**
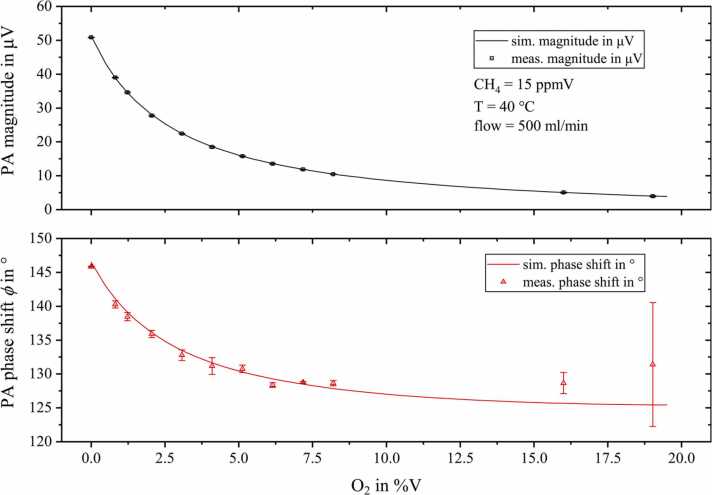
Measured photoacoustic magnitude (black squares, upper graph) and phase shift ϕ (red triangles, lower graph) for 15 ppmV methane diluted in dry nitrogen with rising oxygen content. The calculation results obtained from CoNRad with the energy transitions and reaction rates listed in appendix B, Table B.2 are represent by solid lines.

As soon as oxygen is added to a mixture of methane and nitrogen, an exponential magnitude decrease accompanied by a pronounced phase shift can be observed. At the maximum added O_2_ concentration of 19.03%V, the PA amplitude drops to 7.8% of its initial value. At the same time, a phase shift of approximately 14.4° is observed. The results calculated by CoNRad are plotted as solid lines in Fig. 8, showing excellent agreement with the measurement. A list with all relevant energy transitions can be found in the appendix B, Table B.2.

The relaxation characteristics for this scenario can be explained as follows. The ICL excites $\nu_{{\text{s}}_{1}}$ at 2968 cm^−1^, which relaxes with $k_{1}$ quite fast to the ${2\nu }_{b}$ state. From this state, several energy transitions are possible. Via $k_{2}$ and $k_{5}$ intramolecular energy transitions resulting in $\nu_{b}$ occur. With reaction 4 ($k_{4}$), part of the vibronic energy is transferred to the vibrational state of oxygen $O_{2}(\nu )$. This state can also be excited via reaction 7 ($k_{7}$), which is in direct competition with the classical VT relaxation processes ($k_{12}$) of the $\nu_{b}$ state. Since the intermolecular (VV) energy transitions ($k_{4}$, $k_{7}$) are comparatively fast ($>1\cdot 10^{6}s^{-1}{\mathrm{atm}}^{-1}$, see appendix B, Table B.2), they dominate the relaxational behavior of CH_4_ even at low O_2_ concentrations. Due to the rather slow relaxation process originating from $O_{2}(\nu )$ ($k_{14}$),^3^ a majority of the initially absorbed laser energy accumulates in $O_{2}(\nu )$ and can no longer contribute to photoacoustic signal generation, yielding a magnitude decrease.

All measurements are based on raw data that was averaged over one minute, with a data acquisition rate of 5 Hz and a lock-in time constant of 5 s. The error bars indicate ±3 times the standard deviation of raw data.

### 3.3 The effect of water on the photoacoustic detection of methane

**Fig. 9:**
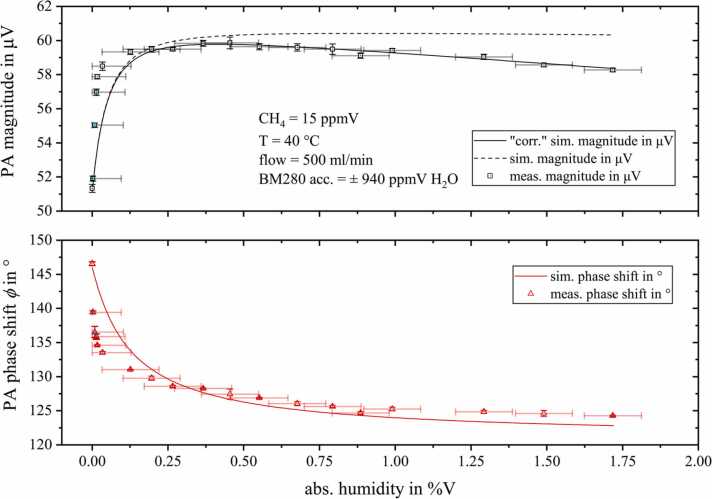
Measured photoacoustic magnitude (black squares, upper graph) and phase shift ϕ (red triangles, lower graph) for 15 ppmV methane diluted in nitrogen with rising humidity content. The calculation results obtained from CoNRad are represent by solid lines.

To further investigate the signal characteristics resulting from water-induced relaxation changes, we integrated a setup that allows trace humidification of the sample (refer to Fig. 3). As the BME280 is not able to monitor traces of water, the first three water concentrations (light blue filled measurement points in Fig. 9) were approximated linearly to the respective measured PA magnitude and to the BME reading of the fourth measuring point. According to the datasheet, the measurement accuracy of the BME280 is specified as ±3% relative humidity at 25 °C. This corresponds to approximately ±940 ppmV H_2_O.

By adding water to the measurement matrix, the exact opposite is achieved compared to adding O_2_. While oxygen inhibits PA signal generation due to extremely slow VT relaxation rates ($k_{14}$), H_2_O causes a significant increase in magnitude (+16.6% at 0.45%V H_2_O, compared to the dry nitrogen). Again, this magnitude change is accompanied by a distinct phase shift, of − 22.3° for 1.72%V H_2_O. This implies, that methane cannot relax completely in pure nitrogen at an acoustic frequency of *f*_res_ = 5205 Hz. Due to the two very fast VV transitions ($k_{3}$, $k_{6}$) from methane to water, the transitions from ${2\nu }_{b}$ and $\nu_{b}$ to ${H_{2}O(\nu }_{2})$ dominate the relaxation of methane. Since excited ${H_{2}O(\nu }_{2})$ itself rapidly relaxes to the ground state via reaction 13 ($k_{13}$), the entire absorbed energy is transferred into kinetic energy contributing to PA signal generation and resulting in a pronounced signal increase by adding small amounts of water.

**Fig. 10:**
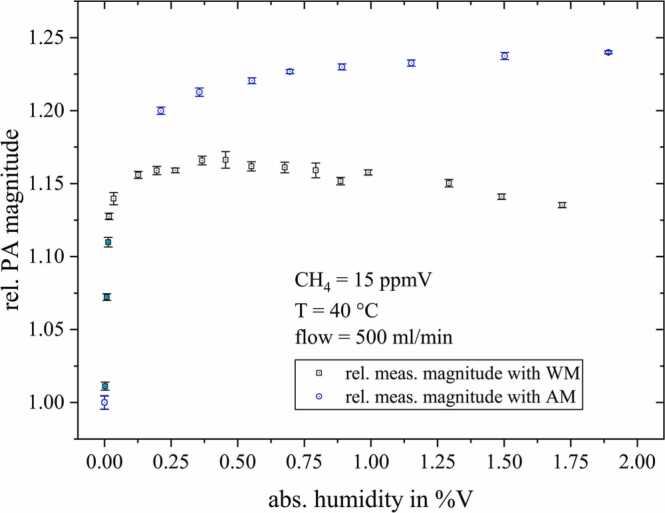
Comparison of the measured relative magnitudes resulting from amplitude modulated (AM, blue circles) and wavelength modulated (WM, black squares) methane detection, normalized to the magnitude measured for dry conditions.

By further adding water, Fig. 9 reveals the photoacoustic magnitude to linearly decrease with $-1.9\%/\%V_{H_{2}O}$. This decay was included as an empirical value in CoNRad as it is assumed to be no relaxation effect. The original simulation without this empirical quantity is displayed as a dashed line in Fig. 9. We confirmed our assumption of a non-relaxation dependent decay of the PA magnitude due to water by repeating this measurement series using amplitude modulation (AM) of the ICL instead of wavelength modulation (WM) (see Fig. 10). Since Fig. 10 reveals this signal decay to only occur when applying WM, we rather assume peak deformations caused by pressure or collisional broadening effects to be responsible.

### 3.4 The combined effect of oxygen and water on the photoacoustic detection of methane

**Fig. 11:**
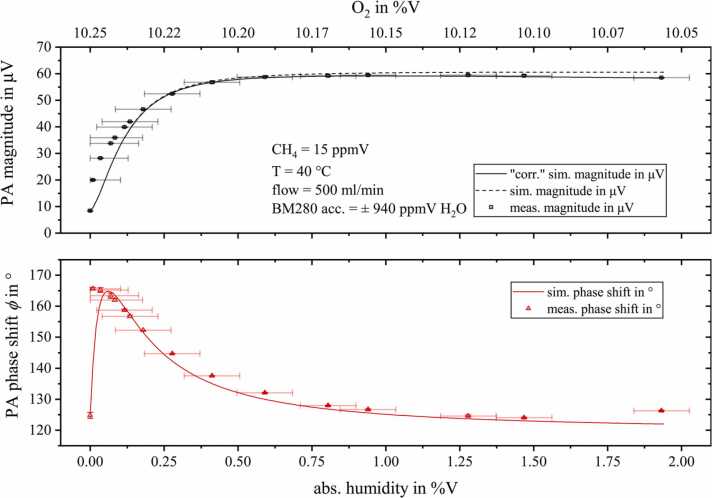
Measured photoacoustic magnitude (black squares, upper graph) and phase shift ϕ (red triangles, lower graph) for 15 ppmV methane diluted in a nitrogen, oxygen mixture with rising humidity content. The calculation results obtained from CoNRad are represent by solid lines.

Starting with a gas matrix of 15 ppmV CH_4_ diluted in a nitrogen (89.75%V), oxygen (10.25%V) mixture and continuously adding water using the setup from Fig. 3 allowed us to investigate the PA signal characteristics in much more detail compared to [9]. As displayed in the upper graph of Fig. 11, the magnitude loss induced by O_2_ is completely compensated by adding water (refer to appendix B, Table B.2, reactions 3, 6, 8 and 13). In total, 29 individual energy transitions were considered by CoNRad for the calculations of this relaxation scenario. The linear magnitude decrease by adding water was observed again and implemented in the calculations (solid line, upper graph in Fig. 11). For very low moisture levels the phase exhibits a sharp shift of about 40.9°. As soon as the slope of the PA magnitude weakens, the phase decreases again and remains almost constant for higher humidity values (>1%V).

### 3.5 Applying CoNRad to other setups

CoNRad allows to quantify relaxation-induced signal changes for arbitrary complex systems. Besides relaxation phenomena, other parameters such as Q-factor, speed of sound or heat capacity ratio might also significantly affect the PA signal. However, as these parameters, in turn, are influenced in a complex way by environmental conditions, i.e. temperature, pressure and gas composition, those correlations as well as their effect on PA signal generation are discussed in a separate work that will be published soon.

Relaxational and non-relaxational effects on the photoacoustic signal are often mixed when interpreting cross-sensitivities which can lead to misinterpretations. Recently, Wu et al. and Elefante et al. developed compact quartz enhanced photoacoustic spectroscopy (QEPAS) sensors for the detection of CH_4_ in humid environments with resonance frequencies of 17.741 kHz and 12.456 kHz, respectively [4], [29]. Both were able to achieve parts per billion level LoDs, i.e. 50 ppbV (1σ) with 1 s integration time [4] and 180 ppbV (1σ) with 0.2 s integration time [29]. Wu et al. and Elefante et al. employed an interband cascade laser (ICL) emitting at 3038.5 cm^−1^ and 2988.8 cm^−1^, respectively. However, they describe a linear increase of the PA amplitude by continuously adding water within a humidity range from approximately 0.38%V up to 1.6%V to a mixture of traces of CH_4_ diluted in N_2_ or laboratory air, respectively. In both publications, this linear correlation was assumed to result from a water-induced acceleration of the VT relaxation behavior of CH_4_.

Simulating these systems with CoNRad reveals a sharp amplitude gain due to water induced acceleration of relaxation, which, however, is already completed for water concentrations of about 0.4%V. Further adding water does not cause any further relaxation-induced increase in PA amplitude. Therefore, we assume this linear signal increase in [4], [29] rather to be attributed to acoustic detuning. As already reported earlier, double-resonant QEPAS systems are known to be prone to acoustic detuning, as the acoustic resonance depends on the speed of sound within the resonator tube, which in turn depends on temperature and composition of the sample, while the resonance frequency of the QTF is hardly affected by temperature and composition [8], [30].

**Fig. 12:**
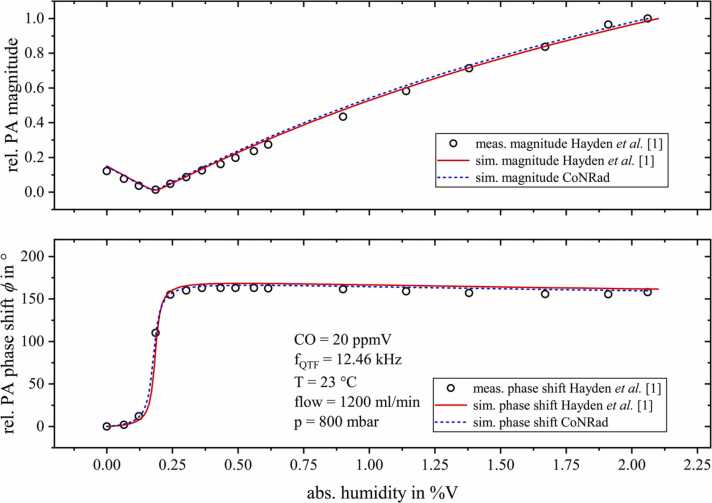
Direct comparison of the relative measured photoacoustic data (black circles) from [1] (upper graphs: magnitude, lower graphs: phase shift) with the simulation results from [1] (red solid lines) and the algorithm (CoNRad) presented in this work (blue dashed lines). The data was measured by Hayden et al. by means of QEPAS based carbon monoxide (CO) detection diluted in nitrogen (N_2_) with varying humidity content.

For the photoacoustic detection of carbon monoxide (CO), by means of QEPAS, in air-like gas matrices, Hayden et al. [1] and Sgobba et al. [14] both developed a theoretical rate equation model describing the complete non-radiative relaxation process of CO in humid nitrogen and humid air. Their theoretical calculations showed excellent agreement with their measurement results. Based on the relaxation rates listed in [1] and [14], we were also able to reproduce the amplitude and phase characteristics of the photoacoustic measurement data reported in [1] (see Fig. 12). This emphasizes the versatile application possibilities of CoNRad.

## 4 Conclusion

In this work we have demonstrated the relevance of correctly modelling the relaxation cascade in view of photoacoustic signal generation. Simplified two-level systems and not accounting for intermolecular energy transitions often fail to adequately describe relaxation-induced signal changes. We presented an autonomous algorithm that is capable of modelling any relaxation cascade, regardless of its complexity, thus providing the basis for calculating photoacoustic signals. This algorithm only requires the individual reactions with their corresponding reaction rates and the energies of the vibrational states as input data. Since literature regarding the reaction rates is often not available, this poses the biggest restriction of the algorithm. However, using this approach, we provide a solid basis for calculating different relaxation effects for any analyte, in different applications. Combining CoNRad with spectral measurements, e.g. performing spectral scans as conducted by Menduni et al., would significantly improve the resilience of photoacoustic sensors towards potential cross-sensitivities, even in complex measurement conditions [31]. This enables the successful transfer of photoacoustic sensor systems from academia to industry.

## Funding

Essential financial support for this work has been provided within the scope of the project PreSEDA funded by the German government and the Federal Ministry of Economic Affairs and Energy (BMWi). The funding code of this project is 03EN2028A.

## Declaration of Competing Interest

The authors declare that they have no known competing financial interests or personal relationships that could have appeared to influence the work reported in this paper.

## Figures and Tables

**Table B.2:** Summary of all collision-based energy transitions (reactions), their energy difference ∆E and respective reaction rate k for the complete de-excitation process during photoacoustic methane detection with regards to gas matrices containing nitrogen, oxygen and water (see Fig. 7).

	Reaction	dE**in cm**^**−1**^	**Reaction rate*****k*****in s**^**−1**^**atm**^**−1**^ **used literature**	
$\left(1\right)$	${\mathrm{CH}}_{4}\left(\nu_{s_{1}}\right)+M\;\overset{k_{1}^{M}}{\to }\;{\mathrm{CH}}_{4}\left(2\nu_{b}\right)+M$	−124	$k_{1}^{{\mathrm{CH}}_{4}}=2.1\cdot 10^{8}$	$2.1\cdot 10^{8}$[32]
			$k_{1}^{N_{2}}=4.6\cdot 10^{8}$	$4.6\cdot 10^{8}$[33]
			$k_{1}^{H_{2}O}=1\cdot 10^{8}$	/
			$k_{1}^{O_{2}}=4.6\cdot 10^{8}$	$4.6\cdot 10^{8}$[33]
$\left(2\right)$	${\mathrm{CH}}_{4}\left(2\nu_{b}\right)+{\mathrm{CH}}_{4}\;\overset{k_{2}}{\to }\;{\mathrm{CH}}_{4}\left(\nu_{b}\right)+{\mathrm{CH}}_{4}\left(\nu_{b}\right)$	0	$k_{2}=5\cdot 10^{8}$	$5.5\cdot 10^{8}$[34]
$\left(3\right)$	${\mathrm{CH}}_{4}\left(2\nu_{b}\right)+H_{2}O\;\overset{k_{3}}{\to }\;{\mathrm{CH}}_{4}\left(\nu_{b}\right)+H_{2}O\left(\nu_{2}\right)$	173	$k_{3}=1\cdot 10^{8}$	/
$\left(4\right)$	${\mathrm{CH}}_{4}\left(2\nu_{b}\right)+O_{2}\;\overset{k_{4}}{\to }\;{\mathrm{CH}}_{4}\left(\nu_{b}\right)+O_{2}\left(\nu \right)$	134	$k_{4}=1\cdot 10^{7}$	$6.6\cdot 10^{6}$[34]
$\left(5\right)$	${\mathrm{CH}}_{4}\left(2\nu_{b}\right)+M\;\overset{k_{5}^{M}}{\to }\;{\mathrm{CH}}_{4}\left(\nu_{b}\right)+M$	−1422	$k_{5}^{{\mathrm{CH}}_{4}}=1.6\cdot 10^{6}$	$1.6\cdot 10^{6}$[35]
			$k_{5}^{N_{2}}=2\cdot 10^{5}$	$1.6\cdot 10^{5}$[35]
			$k_{5}^{H_{2}O}=2.4\cdot 10^{5}$	/
			$k_{5}^{O_{2}}=2.6\cdot 10^{5}$	/
$\left(6\right)$	${\mathrm{CH}}_{4}\left(\nu_{b}\right)+H_{2}O\;\overset{k_{6}}{\to }\;{\mathrm{CH}}_{4}+H_{2}O\left(\nu_{2}\right)$	173	$k_{6}=7\cdot 10^{7}$	/
$\left(7\right)$	${\mathrm{CH}}_{4}\left(\nu_{b}\right)+O_{2}\;\overset{k_{7}}{\to }\;{\mathrm{CH}}_{4}+O_{2}\left(\nu \right)$	134	$k_{7}=2\cdot 10^{6}$	$3.3\cdot 10^{6}$[34]
$\left(8\right)$	$O_{2}\left(\nu \right)+H_{2}O\;\overset{k_{8}}{\to }\;O_{2}+H_{2}O\left(\nu_{2}\right)$	39	$k_{8}=2.2\cdot 10^{7}$	$2.2\cdot 10^{8}$[36]
$\left(9\right)$	$O_{2}\left(\nu \right)+{\mathrm{CH}}_{4}\;\overset{k_{9}}{\to }\;O_{2}+{\mathrm{CH}}_{4}\left(\nu_{b}\right)$	−134	$k_{9}=3.3\cdot 10^{7}$	$3.3\cdot 10^{7}$[34]
$\left(10\right)$	$H_{2}O\left(\nu_{2}\right)+{\mathrm{CH}}_{4}\;\overset{k_{10\;}}{\to }\;H_{2}O+{\mathrm{CH}}_{4}\left(\nu_{b}\right)$	−173	$k_{10}=2\cdot 10^{7}$	/
$\left(11\right)$	$H_{2}O\left(\nu_{2}\right)+O_{2}\;\overset{k_{11\;}}{\to }\;H_{2}O+O_{2}\left(\nu \right)$		$k_{11}=2\cdot 10^{7}$	$3.8\cdot 10^{7}$[37]
$\left(12\right)$	${\mathrm{CH}}_{4}\left(\nu_{b}\right)+M\;\overset{k_{12\;}^{M}}{\to }\;{\mathrm{CH}}_{4}+M$	−1422	$k_{12}^{{\mathrm{CH}}_{4}}=8\cdot 10^{5}$	$8\cdot 10^{5}$[34]
			$k_{12}^{N_{2}}=4.5\cdot 10^{4}$	$8\cdot 10^{4}$[35]
			$k_{12}^{H_{2}O}=1.2\cdot 10^{5}$	/
			$k_{12}^{O_{2}}=7\cdot 10^{4}$	$1.4\cdot 10^{5}$[34]
$\left(13\right)$	$H_{2}O\left(\nu_{2}\right)+M\;\overset{k_{13\;}^{M}}{\to }\;H_{2}O+M$	−1595	$k_{13}^{{\mathrm{CH}}_{4}}=1\cdot 10^{6}$	/
			$k_{13}^{N_{2}}=1\cdot 10^{7}$	$1\cdot 10^{6}$[36]
			$k_{13}^{H_{2}O}=1.2\cdot 10^{9}$	$1.2\cdot 10^{9}$[38]
			$k_{13}^{O_{2}}=1\cdot 10^{6}$	$1\cdot 10^{6}$[36]
$\left(14\right)$	$O_{2}\left(\nu \right)+M\;\overset{k_{14\;}^{M}}{\to }\;O_{2}+M$	−1556	$k_{14}^{{\mathrm{CH}}_{4}}=1\cdot 10^{6}$	$1\cdot 10^{6}$[39]
			$k_{14}^{N_{2}}=18$	18[40]
			$k_{14}^{H_{2}O}=1.1\cdot 10^{6}$	$8\cdot 10^{5}$[36]
			$k_{14}^{O_{2}}=81$	81[38]

## Appendix B

See Table B.2.

## Appendix A

The total heat production rate $\dot{H}(t)$ of any system can be described as the sum of all individual heat rates released from the number *n* of all involved energy states. This total heat production rate can be directly linked to the photoacoustic pressure $p_{a}$.

(A.1) $$\dot{H}\left(t\right)=\sum_{i}^{n}{\dot{H}}_{i}\left(t\right)$$

with

(A.2) $${\dot{H}}_{i}\left(t\right)=\left[\nu_{i}\right]\left(t\right)\cdot \left[hc_{0}\cdot \left(\sum_{j}^{m}k_{i,j}\cdot \left({\bar{\nu }}_{i}-{\bar{\nu }}_{j}\right)\right)\right]$$

According to Hunter et al. the population densities of every energy state involved in the relaxation process of any given system can be computed according to [26]:

(A.3) $$\left[\nu_{i}\right](t)=\rho_{A}\sigma_{A}\left({\bar{\nu }}_{\mathrm{Ph}}\right)\psi_{0}\tau_{i}\frac{1}{\sqrt{1+{\left(\omega \tau_{v_{i}}\right)}^{2}}}e^{-i\phi_{i}}\left\{\sum_{\begin{array}{c} \mathrm{all}\;\mathrm{routes}\\ \mathrm{to}\;\nu_{i} \end{array}}\left[\prod_{n}^{i-1}\left(\frac{k_{n\to r}}{\sum k_{n}}\frac{1}{\sqrt{1+{\left(\omega \tau_{n}\right)}^{2}}}e^{-i\phi_{n}}\right)\right]\right\}e^{-i\omega t}$$

The product of photon flux $\psi_{0}$, volume number density $\rho_{A}$ of the analyte A and absorption cross section $\sigma_{A}\left({\overset{®}{\nu }}_{\mathrm{Ph}}\right)$ accounts for the initial quanta of absorption per second and per unit volume in s^−1^m^−3^. This quantity acts as a starting point for the relaxation process*.* The sum in equation (9) adds up all individual relaxation routes starting from the initially excited state and resulting in the energy state $\nu_{i}$ that is considered. The ratio $\left(k_{n\to r}/\sum k_{n}\right)$ considers the fractions of molecules which undergo energy transition from the n-state to the r-state $k_{n\to r}$, before reaching the desired state $\nu_{i}$, with regards to all other transitions possibly performed by the n-state $\sum k_{n}$.

## Footnotes

2 A detailed formula for the time dependent population density of any excite state $\left[\nu_{i}\right](t)$ can be found in the appendix A, eq. A.3.

3 In these considerations the VV transition from $O_{2}(\nu )$ to $\nu_{b}$ ($k_{9}$) is neglected due to the low CH_4_ concentrations, yielding very unlikely oxygen – methane collisions. However, CoNRad considers this transition as well.
